# Case report: Non-Alzheimer's disease tauopathy with logopenic variant primary progressive aphasia diagnosed using amyloid and tau PET

**DOI:** 10.3389/fneur.2022.1049113

**Published:** 2022-11-15

**Authors:** Yuki Momota, Mika Konishi, Keisuke Takahata, Taishiro Kishimoto, Toshiki Tezuka, Shogyoku Bun, Hajime Tabuchi, Daisuke Ito, Masaru Mimura

**Affiliations:** ^1^Department of Neuropsychiatry, Keio University School of Medicine, Tokyo, Japan; ^2^Department of Functional Brain Imaging Research, National Institute of Radiological Sciences, National Institutes for Quantum and Radiological Science and Technology, Chiba, Japan; ^3^Psychiatry Department, Donald and Barbara Zucker School of Medicine, New York, NY, United States; ^4^Department of Neurology, Keio University School of Medicine, Tokyo, Japan; ^5^Department of Physiology/Memory Center, Keio University School of Medicine, Tokyo, Japan

**Keywords:** logopenic variant, primary progressive aphasia, tauopathy, Alzheimer's disease, frontotemporal lobar degeneration, positron emission tomography

## Abstract

We report a patient with logopenic variant primary progressive aphasia (lv-PPA) who was diagnosed as having non-Alzheimer's disease (AD) tauopathy after multiple biophysical/biological examinations, including amyloid and ^18^F-florzolotau tau positron emission tomography (PET), had been performed. A woman in her late 60s who had previously been diagnosed as having AD was referred to us for a further, detailed examination. She had been unaware of any symptoms at the time of AD diagnosis, but she subsequently became gradually aware of a speech impairment. She talked nearly completely and fluently, although she occasionally exhibited word-finding difficulty and made phonological errors during naming, word fluency testing, and sentence repetition; these findings met the criteria for the diagnosis of lv-PPA, which is known to be observed more commonly in AD than in other proteinopathies. Magnetic resonance imaging, single photon emission computed tomography, and plasma phosphorylated tau and plasma neurofilament light chain measurements showed an AD-like pattern. However, both ^11^C-Pittsburgh compound-B and ^18^F-florbetaben amyloid PET showed negative results, whereas ^18^F-florzolotau tau PET yielded positive results, with radio signals predominantly in the left superior temporal gyrus, middle temporal gyrus, supramarginal gyrus, and frontal operculum. Whole-genome sequencing revealed no known dominantly inherited mutations in AD or frontotemporal lobar degeneration genes, including the genes encoding amyloid precursor protein, microtubule-associated protein tau, presenilin 1 and 2. To the best of our knowledge, this patient was a rare case of lv-PPA who was diagnosed as having non-AD tauopathy based on the results of multiple examinations, including whole-genome sequencing, plasma measurement, and amyloid and ^18^F-florzolotau tau PET. This case underscores the clinicopathologically heterogeneous nature of this syndrome.

## Introduction

Primary progressive aphasia (PPA) is a neurodegenerative syndrome that is known to be associated with both Alzheimer's disease (AD) and frontotemporal lobar degeneration (FTLD), which is characterized by progressive language impairment as the most salient clinical feature and is commonly associated with a selective lesion in the perisylvian region of the left hemisphere (1, 2). Logopenic variant (lv-)PPA is a syndrome characterized by fluent speech and impaired sentence repetition and sentence comprehension, resembling vascular conduction aphasia (3); the most frequent cause is AD (4–6), while less than 20% of cases are found to have FTLD-tau (6, 7).

The clinical characteristics of AD-related proteinopathies are often similar, but they are concurrently heterogeneous in every patient, complicating diagnosis (8, 9). From this viewpoint, genetic and molecular biomarkers could provide better clues to the underlying pathology. Among the known genetic markers, presenilin 1 (*PSEN1*)/*PSEN2* and amyloid precursor protein (*APP*) variants can be observed in cases with lv-PPA, while chromosome 9 open reading frame 72 (*C9orf72)*, microtubule-associated protein tau (*MAPT*), and some progranulin (*GRN*) mutations have been reported in non-fluent/agrammatic variant (nfv-)PPA cases, and GRN/TAR DNA-binding protein of 43 kDa (TDP-43) has been reported in semantic variant (sv-)PPA cases (2). The plasma levels of phosphorylated tau (p-tau) 181 are elevated in AD (10), whereas the plasma levels of neurofilament light chain protein (NFL) are elevated in both AD and FTLD (11), although the levels are higher in FTLD (12). Positron emission tomography (PET), particularly tau PET, enables visual observation of the deposited causative proteins in a region-specific manner (13).

While the clinicopathological relationships in lv-PPA have remained somewhat unclear, recent studies have described the clinical characteristics of cases with atypical heterogeneous lv-PPA, as well as those of autopsy-confirmed cases of AD with lv-PPA, which have promoted a better understanding of the syndrome (5–7, 14–18). For example, approximately one-third of patients with lv-PPA may have cerebral microbleeds and superficial siderosis (16, 17); in rare instances, patients with lv-PPA may have GRN mutations (18).

Herein, we report a patient with lv-PPA who, despite an initial clinical diagnosis of AD, was suspected of having non-AD tauopathy. To the best of our knowledge, this patient represents an exceptional example of lv-PPA (19) in whom the pathological basis was difficult to predict even after multiple examinations including genome sequencing, plasma p-tau181 and NFL examinations, ^11^C-Pittsburgh Compound-B (PiB) and ^18^F-florbetaben (FBB) amyloid PET (20, 21), and ^18^F-florzolotau, i.e., ^18^F-PM-PBB3 (propanol modification of pyridinyl-butandienyl-benzothiazole 3) tau PET (22). We hope that this report provides further insight into the correlations among clinical symptoms, biomarkers, and brain imaging findings in proteinopathies, paving the way for early diagnosis and novel therapies for this disease entity.

## Case report

A woman in her late 60s was referred to our hospital for a detailed examination of her language impairment. She was right-handed and had more than 16 years of education. Two years previous to her visit to our hospital, she had been referred to a dementia specialist after the Mini-Mental State Examination (MMSE) performed while she was hospitalized for hypertension revealed mild dementia-level scores. She was diagnosed as having AD, taking into consideration that fluorodeoxyglucose (FDG) PET demonstrated hypometabolism in her left medial temporal lobe, posterior cingulate gyrus, and precuneus; ^11^C-Pittsburgh Compound-B (PiB) amyloid PET yielded a positive plausible result with marginal tracer accumulation in the white matter and partial accumulation in the parietal and lateral temporal lobes; the mean standard uptake value ratio (MSUVR) on PiB amyloid PET was 1.36, which was slightly lower than a previously reported cutoff value of 1.50 (23). She was unaware of any cognitive decline, including memory impairment, at the time of the diagnosis, but she subsequently became gradually aware of a language impairment. Another doctor was asked for a second opinion, and she was referred to our hospital based on a suspicion of primary progressive aphasia with a pathological basis of FTLD (or atypical AD).

Her chief complaint was stagnation of speech, especially when she was nervous. Her husband also told us that she sometimes mispronounced words while reading aloud. She had a professional career and had no remarkable problems at work. She was aware of an age-appropriate memory decline but had no obvious subjective memory complaints. Her past medical history included hypertension and coxarthrosis. She had no family history of dementia, stroke, or other neurodegenerative diseases.

### Neurological findings

No obvious motor symptoms, pyramidal/extrapyramidal symptoms, or ataxia were observed. She looked cheerful, and she talked sociably and nearly completely and fluently without obvious apraxia of speech or paraphasia, although word-finding difficulty was occasionally observed during brief object naming and word fluency tasks. She was able to remember her daily events. Her episodic and semantic memory seemed to be well maintained.

### Neuropsychological test findings

Neuropsychological tests suggested mild to moderate impairment in language and verbal short-term memory (Table 1): her MMSE score (24) was 23/30; her Wechsler Memory Scale-Revised (WMS-R) Logical Memory score (25) was 7/25 for the immediate recall and 3/25 for the delayed recall; and her Rey-Osterrieth Complex Figure Test score (26, 27) was 36/36 for the copy and 14/36 for the 3-min delayed recall. An assessment using the Japanese Standard Language Test of Aphasia (28, 29) suggested marginal to mild overall language impairment, particularly in oral expressions, where word-finding difficulty and/or phonological errors were observed in naming and sentence repetition; the findings also suggested impaired auditory comprehension of not words, but sentences (e.g., Sequential Commands) (Supplementary Table 1). Similarly, in the sentence repetition task in the MMSE, she correctly repeated the first phrase and the first syllable of the subsequent phrase, but she could not continue thereafter. After receiving a clue for the first two syllables, she was able to continue the phrase correctly, although she failed to complete the last phrase, for which she substituted completely different words from those used in the original sentence.

**Table 1:** Neuropsychological test scores.

		**One-year**
	**Initial visit**	**follow-up**
Mini-mental state examination	23	22/30
Raven's colored progressive matrices	32	N/A/36
Rey-Osterrieth complex figure test		
(Copy)	36	N/A/36
(3-min delayed)	14	N/A/36
Logical memory		
(Immediate)	7	7/25
(Delayed)	3	3/25
Rey auditory verbal learning test		
Trial 1	2	N/A/15
Trial 2-3-4	4-5-3	N/A/15
Trial 5	4	N/A/15
Interference list B	3	N/A/15
Trial 6	3	N/A/15
Recognition	15	N/A/15
Word fluency		
(Category)	31	20
(Initial letter)	25	19

N/A represents tests that were not administered.

An examination performed 1 year after her initial visit to our hospital showed a notable decline in the Word Fluency (3 min) score only (Category: 20, Initial letter: 19) (Table 1). In the sentence repetition task in the MMSE, she correctly repeated the first and the last phrases but omitted the two phrases in the middle. Three more tests were additionally performed at this time. On the Japanese version of the Alzheimer's Disease Assessment Scale-Cognitive subscale (ADAS-cog-J) (30), she scored 11.4/70 and exhibited phonological errors involving the replacement, omission or insertion of syllables [e.g., *“ki-me-tsu-ri*” instead of *“tsu-me-ki-ri*” (i.e., nail cutter), “*o-yu-bi*” for “*o-ya-yu-bi*” (i.e., thumb), “*ko-ya-yu-bi*” for “*ko-yu-bi*” (i.e., pinky); Supplementary Table 2]. On the Japanese Adult Reading Test (JART) (31), she scored 5/50 (equivalent to a predicted IQ of 81): three words with highly irregular readings (e.g., tobacco) were not scored after she answered using a gesture and/or explanation, 25 words were incorrect or partially correct, and 17 words were unanswered. Her Clinical Dementia Rating (CDR) score (32) was 0.5, and she was continuing to work as before without experiencing any remarkable problem. No obvious grammatical errors were observed in the above-mentioned assessments.

### Clinical diagnosis

Based on the clinical findings, i.e., almost completely fluency speaking (except for slight language impairment in the form of word-finding difficulty and phonological errors), impairment in verbal short-term memory, absence of obvious cognitive decline in other domains including visual or episodic memory, and absence of motor and pyramidal/extrapyramidal symptoms, or ataxia, the most likely clinical diagnosis was lv-PPA with a questionable pathological basis of AD according to the criteria for lv-PPA (33).

### Brain imaging

**Figure 1:**
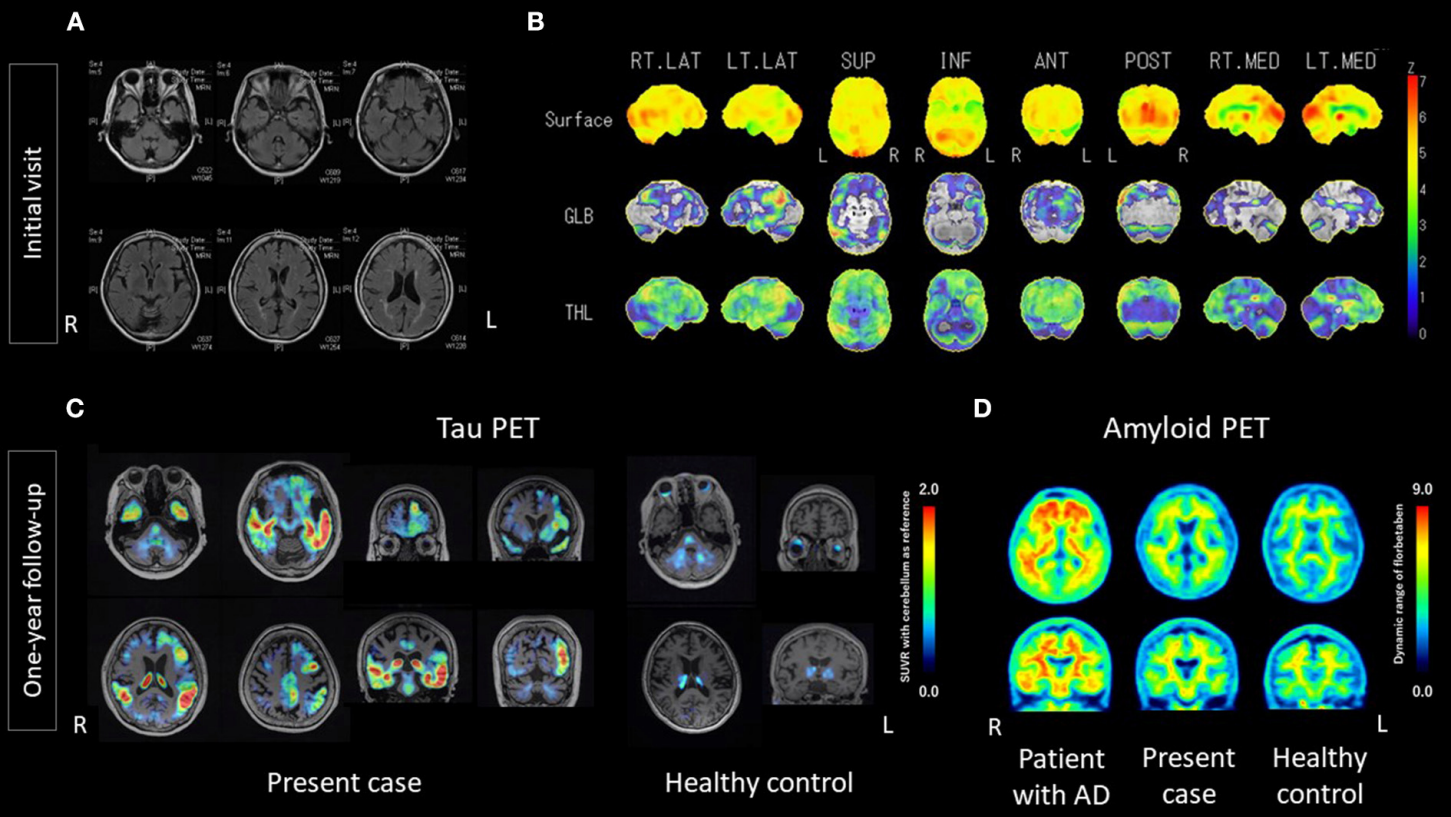
Results of MRI, 3D-SSP of SPECT, ^18^F-florzolotau tau PET, and ^18^F-florbetaben amyloid PET. **(A)** MRI at the initial visit showed mild atrophy of the cerebrum, with a left predominance, and of the cerebellum. Hyperintense signals in the white matter suggest old lacunar infarctions and/or chronic ischemic changes. **(B)** 3D-SSP of SPECT at the initial visit showed a mild decrease in blood flow in the posterior cingulate gyrus, precuneus and cerebellum. **(C)** ^18^F-florzolotau tau PET at one-year follow-up showed intense radio signals predominantly in the left temporal lobe, particularly the superior temporal and middle temporal lobe, as well as the supramarginal gyrus, and marginal to mild signals in the frontal lobe. **(D)** ^18^F-florbetaben amyloid PET at one-year follow-up did not show intense radio signals, compared with age/sex-matched controls. AD, Alzheimer's disease; ANT, anterior; GLB, global; INF, inferior; L; left; LT, left; MED, medial; POST, posterior; R, right; RT, right; SUP, superior; SUVR, standard uptake value ratio; THL, thalamus; 3D-SSP, Three-dimensional stereotactic surface projections.

At the time of the patient's first visit to our hospital, visual assessments of magnetic resonance imaging (MRI) findings showed atrophy, particularly in the left temporal lobe and cerebellum; fluid-attenuated inversion recovery (FLAIR) imaging showed high signals in the white matter, suggesting old lacunar infarctions and/or chronic ischemic changes (Figure 1A). Single photon emission computed tomography (SPECT) showed left-predominant hypoperfusion in the parietal lobes and the left temporal lobe and mild hypoperfusion in both frontal lobes. A statistical analysis using 3D-stereotactic surface projections (3D-SSP) showed a mild decrease in blood flow in the posterior cingulate gyrus, the precuneus and the cerebellum (Figure 1B).

One year after the initial visit, visual assessments of MRI findings showed no remarkable changes, compared with the previous imaging findings. ^18^F-florbetaben (FBB) amyloid PET (21) was negative, as judged by certified radiologists. However, ^18^F-florzolotau tau PET (22) was positive, as judged by trained neurologists and psychiatrists. The accumulations of ^18^F-florzolotau were predominantly on the left side, particularly in the superior temporal gyrus, middle temporal gyrus, supramarginal gyrus, and frontal operculum (Figure 1C; see Supplementary Figure 1 for more details). Volume of interest (VOI) analyses using FreeSurfer 6.0 (http://surfer.nmr.mgh.harvard.edu/) from the Desikan-Killiany-Tourville atlas (34) demonstrated that the SUVRs in the present case were higher than those of healthy controls, with *z*-scores of 27.37, 10.95, 13.79, and 34.76 for the supramarginal, inferior-temporal, middle-temporal, and superior-temporal gyrus, respectively (Supplementary Table 3).

Positron emission tomography imaging acquisition, processing, and assessment were conducted as follows. ^18^F-florbetaben amyloid PET images were acquired for 20 min using PET-CT (True Point Biograph 40/64; Siemens Japan K.K., Tokyo, Japan) at 90 min after the intravenous injection of 300 MBq ± 10% ^18^F-florbetaben. The 20-min PET images were interpreted by two nuclear medicine experts who had completed a training program offered by the manufacturer (Piramal Imaging GmbH, Berlin, Germany). Following the NeuraCeq™ guidelines, amyloid-β positivity or negativity was determined based on assessments of tracer uptake in the gray matter in the following four brain regions: the lateral temporal lobes, the frontal lobes, the posterior cingulate cortex/precuneus, and the parietal lobes (http://www.accessdata.fda.gov/drugsatfda_docs/label/2014/204677s000lbl.pdf) (35). Amyloid-β negativity was established when the tracer uptake (i.e., signal intensity) in the gray matter was lower than that in the white matter in all four brain regions. ^18^F-florzolotau tau PET images were acquired for 20 min using PET-CT (Biograph mCT flow, Siemens, Munich, Germany) at 90 min after the intravenous injection of 185MBq ± 10% ^18^F-florzolotau. We used PMOD software (PMOD Technologies, Zürich, Switzerland) to process the 20-min PET images, and tau positivity or negativity was determined based on assessments of tracer uptake using SUVR with reference to the cerebellum. ^18^F-FBB amyloid PET images with dynamic range are shown in Figure 1D.

### Plasma measurements

One year after the initial visit to our hospital, the plasma p-tau181 and NFL levels were measured using the commercial Quanterix^®^ assay (Simoa^®^ p-Tau181 Advantage Kit or Simoa^®^ NF-light Kit) on an HD-1 analyzer or SR–X, in accordance with the respective manufacturer's instructions (Quanterix). The plasma level of p-tau181 was 2.99 pg/ml, while that of NFL was 22.71 pg/ml. These levels suggested an AD-like pattern when they were compared with preliminary cutoff values based on our in-lab data (2 pg/ml for p-tau181 and 35 pg/ml for NFL), although no universal cutoff values have been established (11).

### Whole-genome sequencing

One year after the initial visit to our hospital, genomic DNA was extracted using the DNeasy Blood and Tissue kit (Qiagen). The extracted DNA was amplified by polymerase chain reaction (PCR) using primers designed specifically for target single nucleotide polymorphisms (SNP). Whole-genome sequencing revealed no known dominantly inherited mutations in the AD or FTLD genes, including *APP*, charged multivesicular body protein 2B (*CHMP2B*), *GRN, MAPT, PSEN1, PSEN2*, progranulin (*PGRN*)*, TDP43*, and valosin-containing protein (*VCP*).

## Discussion

Based on the clinical findings, particularly the negative results of amyloid PET and the positive results of tau PET, and the contradictory results of the plasma measurements, the present patient was considered to be a rare case of non-AD tauopathy with lv-PPA, with an underlying pathology that was difficult to predict.

Her language symptoms were considered typical of lv-PPA (4, 6), meeting all the features described in the widely accepted current criteria for the clinical diagnosis of lv-PPA (33): “impaired single-word retrieval in spontaneous speech and naming” and “impaired repetition of sentences and phrases” as the core features, and “speech (phonologic) errors in spontaneous speech and naming,” “spared single-word comprehension and object knowledge,” “spared motor speech,” and “absence of frank agrammatism defined as the omission and/or substitution of grammatical morphemes with associated grammatical errors (36)” as non-core features.

The conspicuous tau PET tracer accumulations, which were predominantly in the left supramarginal/angular gyrus (Figure 1C), seemed to be consistent with the regional brain function and the manifested symptoms in the present case. In particular, tau PET tracer accumulations in the posterior temporal lobe and inferior parietal lobe (supramarginal/angular gyrus) may be the underlying neural basis for the “logopenic” status, which is explained by the dysfunction of the “phonological loop,” a component of short-term memory that includes a store in which phonological memory traces are held over a period of a few seconds, and an articulatory rehearsal process that refreshes them (3). The impairment of the “phonological loop,” which is generally well correlated with AD pathology (6, 14), seemed to have manifested in our patient as syllabic errors in naming and reading aloud, incomplete sentence repetition, and impaired auditory comprehension of sentences. For example, as also described in the Results section, she was able to repeat the first two or three words/morphemes in the sentence repetition task of the MMSE correctly, but she failed to complete subsequent parts because of simplifications or substitutions; in the SLTA, errors were observed in sentence-level auditory comprehension, despite spared word-level auditory comprehension and sentence-level reading comprehension; in the JART, she answered with gestures or a roundabout explanation for some kanji words with highly irregular readings, suggesting that she knew the meaning of the words, but could not find the proper words and/or phonological representation (i.e., how to read the words aloud). The same processes were assumed to account for most of the remaining unscored words. For these reasons, her predicted IQ of 81 (5/50 correct answers) was likely an underestimation caused by her verbal-predominant cognitive decline arising from disease-caused language impairment. Accordingly, the elements for a clinical diagnosis of lv-PPA based on the current diagnostic criteria (33) were applicable in this single case, even though the elements for an imaging-supported diagnosis or a diagnosis with a definite pathology were not present.

A decisive diagnosis based on the positive tau PET findings would be speculative, since ^18^F-florzolotau does not discriminate among the subtypes of tau isoforms [i.e., 3-repeat (3R), 4-repeat (4R), and a mixture of 3- and 4-repeat (3R + 4R) isoforms]. Nevertheless, the diagnostic likelihood could be considered as follows. The most common and important differential diagnosis would be other 3R + 4R tauopathies, such as primary age-related tauopathy (PART), including senile dementia of the neurofibrillary tangle type (SD–NFT) without amyloid plaques; however, the clinical findings lacked the distinctive features of PART, namely, an obvious memory decline, a late onset (i.e., late-80s), and the characteristic limitation of tau lesions to the medial temporal lobe (37). In addition, the findings of tau PET imaging in the present case may not necessarily be PART-like, since the radio signals of ^18^F-florzolotau were seen in the left superior and middle temporal gyrus, left supramarginal gyrus, and left frontal operculum, whereas those in the preclinical stage of AD or PART may expand from the medial temporal cortex, involving less-mature tau fibrils, to the other neocortical and limbic areas, along with the progression of the NFT stage (22). Four-repeat tauopathies such as corticobasal degeneration might be plausible, based on the asymmetric distribution patterns on tau PET imaging, despite not presenting with a typical corticobasal degeneration or progressive supranuclear palsy pattern (22), since pyramidal/extrapyramidal symptoms can appear after cognitive decline (38). The absence of behavioral deficits due to frontal lobe dysfunction and characteristic brain atrophy on MRI such as knife-blade atrophy, suggests that Pick's disease is unlikely. Furthermore, ^18^F-florzolotau distribution predominantly in the left supramarginal/angular gyrus is not consistent with three-repeat tauopathies (22).

In short, most of the biophysical and biological examinations (i.e., MRI, SPECT, FDG PET, and plasma p-tau and NFL measurements) showed an AD-like pattern consistent with the initial clinical diagnosis of AD. In contrast, amyloid PET using both ^11^C-PiB and ^18^F-FBB showed marginal-to-negative results. An ^18^F-florzolotau tau PET and genome sequencing were informative, but the results were inconclusive. No known dominantly inherited mutations of AD or FTLD genes were identified. Notably, AD associated with the *APP* Osaka mutation E693Δ (39, 40) and the Arctic mutation E693G (41), which result in a markedly low amyloid PET retention, was ruled out because no known *APP* mutations were identified.

The above interpretations need to be understood in the context of the following issues. First, although ^18^F-florzolotau shows improved selectivity for tau proteins, including autopsy-confirmed binding to tau proteins in FTLD-tau (22), and does not bind to monoamine oxidase (MAO)-A or MAO-B nor does it cross-react with amyloid-β (22), the possibility of nonspecific/off-target binding should still be considered. Since ^18^F-florzolotau accumulates in the choroid plexus in healthy subjects, some type of off-target binding may exist in this region. Furthermore, in a recent report, the increased retention of ^18^F-florzolotau was found in the basal ganglia of patients with multiple system atrophy, suggesting that cross-reaction with α-synuclein cannot be completely ruled out (42). Second, some potential assessments were not performed: although PET with ^18^F-florzolotau can discriminate a wide range of tauopathies by the pattern of retention, a head-to-head comparison of ^18^F-florzolotau with another tau PET tracer that hardly binds to 4R tau, such as ^18^F-MK-6240 (43), or the dopamine transporter (DAT) imaging and/or ^123^I-metaiodobenzylguanidine (MIBG) scintigraphy (44, 45), might be helpful for a differential diagnosis; it might be desirable to perform a forward digit span, as this task can be sensitive to impairments of the “phonological loop” (6). Third, positivity/negativity on the ^18^F-FBB amyloid PET was determined based only on visual interpretations by certified radiologists. Although our judgmental standards agree with the established guidelines, a quantitative analysis would aid the interpretation and comparison of results. This issue should be pressed forward for future work, while quantitative measures such as the Centiloid (CL) scale, which may allow a direct comparison of results even across different PET tracers, scanning facilities, or analytical methods, are being standardized (46, 47). For the above reasons, long-term follow-up and pathological evaluations might lead to a more precise diagnosis and a better understanding of the clinicopathological basis.

To conclude, we have reported a patient with suspected non-AD tauopathy who presented with lv-PPA and had impairments in naming and sentence repetition as well as verbal short-term memory. Clinical examinations, including MRI, SPECT, FDG-PET, and plasma measurements, showed results compatible with a diagnosis of AD, whereas the amyloid PET yielded mainly negative results and the results of both tau PET and genome sequencing were inconclusive. Since an antemortem diagnosis of proteinopathies is often difficult, we consider the present case to be important from the viewpoint of obtaining a better understanding of proteinopathies, particularly for the collation of clinical symptoms and biological/biophysical findings.

## Data availability statement

The original contributions presented in the study are included in the article/Supplementary material, further inquiries can be directed to the corresponding author/s.

## Ethics statement

The studies involving human participants were reviewed and approved by Certified Review Board of Keio, Keio University School of Medicine. The patients/participants provided their written informed consent to participate in this study.

## Author contributions

YM: conceptualization, resources, investigation, and writing—original draft preparation. MK, KT, TT, and SB: resources and writing—review and editing. TK: writing—review and editing. HT and DI: resources, project administration, and writing—review and editing. MM: resources, writing—review and editing, project administration, funding acquisition, and supervision. All authors contributed to the article and approved the submitted version.

## Funding

This work was supported by the Japan Agency for Medical Research and Development under Grant Number JP17pc0101006 to MM.

## Conflict of interest

The authors declare that the research was conducted in the absence of any commercial or financial relationships that could be construed as a potential conflict of interest.

## Publisher's note

All claims expressed in this article are solely those of the authors and do not necessarily represent those of their affiliated organizations, or those of the publisher, the editors and the reviewers. Any product that may be evaluated in this article, or claim that may be made by its manufacturer, is not guaranteed or endorsed by the publisher.
