# Supplementary material for: Case report: Non-Alzheimer's disease tauopathy with logopenic variant primary progressive aphasia diagnosed using amyloid and tau PET
Source: Front Neurol. 2022 Nov 15;13:1049113. doi: 10.3389/fneur.2022.1049113 (PMC9705984; doi:10.3389/fneur.2022.1049113)
Supplement: Supplementary file 1 [file Data_Sheet_1.pdf]

Supplementary Material

Supplementary Table 1 The Japanese Standard Language Test of Aphasia

Auditory Comprehension		
Words	10	/10
Sentences	9	/10
Sequential Commands	7	/10
Monosyllables	10	/10
Speech		
Picture Naming	17	/20
Word Repetition	10	/10
Action Naming	10	/10
Picture Description (Four-frame Cartoon)	4	/6
Sentence Repetition	3	/5
Animal Category Fluency	7	/15
Reading Aloud		
Kanji Words	5	/5
Kana Letters (Monosyllabic)	10	/10
Kana Words	5	/5
Sentences	5	/5
Reading Comprehension		
Kanji Words	10	/10
Kana Words	10	/10
Sentences	9	/10
Sequential Commands	10	/10
Spontaneous Writing		
Kanji Words	5	/5
Kana Words	5	/5
Picture Description (Four-frame Cartoon)	5	/6
Dictation		
Kana Letters (Monosyllabic)	10	/10
Kanji Words	5	/5
Kana Words	5	/5
Sentences	4	/5
Calculation		
Four Arithmetic Operations	14	/20

Supplementary Table 2 The Japanese version of the Alzheimer's Disease Assessment Scale-Cognitive subscale (ADAS-cog-J)

Total	11	/70
Word Recall	5.7	/10
Commands	1	/5
Constructional Praxis	0	/5
Naming Objects and Fingers	2	/5
Ideational Praxis	0	/5
Orientation	0	/8
Word Recognition	0.7	/12
Remembering Word Recognition Test Instructions	0	/5
Language		
Spoken Language Ability	1	/5
Word Finding Difficulty	0	/5
Comprehension of Spoken Language	1	/5

Supplementary Table 3 Comparison of ^{18}F-florzolotau SUVRs between the present case and those of age-matched healthy controls

Case	Sex	Age	SUVR			
			Supramarginal Gyrus	Inferior-temporal Gyrus	Middle-temporal Gyrus	Superior-temporal Gyrus
Present case (lv-PPA)			2.08	1.77	1.78	2.44
HC1			0.79	0.94	0.81	0.77
HC2			0.65	0.77	0.73	0.65
HC3			0.69	0.89	0.78	0.66
HC4			0.68	0.84	0.81	0.69
HC5			0.70	0.90	0.81	0.69
HC6			0.76	0.99	0.94	0.76
HC (Mean ± SD)	M : F = 3 : 3	70 ± 5.3	0.71 ± 0.05	0.89 ± 0.08	0.81 ± 0.07	0.70 ± 0.05
z-score			27.37	10.95	13.79	34.76

Regional standardized uptake value ratios were calculated using the cerebellar cortex as a reference. F, female; HC, healthy control; lv-PPA, logopenic variant primary progressive aphasia; M, Male; SD, standard deviation; SUVR, standardized uptake value ratio.

Supplementary Figure 1 ^{18}F-florzolotau tau PET

^{18}F-florzolotau tau PET showed intense radio signals predominantly in the left temporal lobe, particularly the superior temporal and middle temporal lobe and the supramarginal gyrus, and marginal to mild signals in the frontal, parietal and occipital lobes. In the basal ganglia, weak signals were observed in the thalamus, subthalamic nucleus, and left putamen. No specific signals were observed in the cerebellum, either in the dentate nucleus or the cerebellar cortex. The strong signals in the choroid plexus may represent non-specific binding. SUVR, standardized uptake value ratio.
