# Supplementary figures and images for: Case report: Non-Alzheimer's disease tauopathy with logopenic variant primary progressive aphasia diagnosed using amyloid and tau PET
Source: Front Neurol. 2022 Nov 15;13:1049113. doi: 10.3389/fneur.2022.1049113 (PMC9705984; doi:10.3389/fneur.2022.1049113)

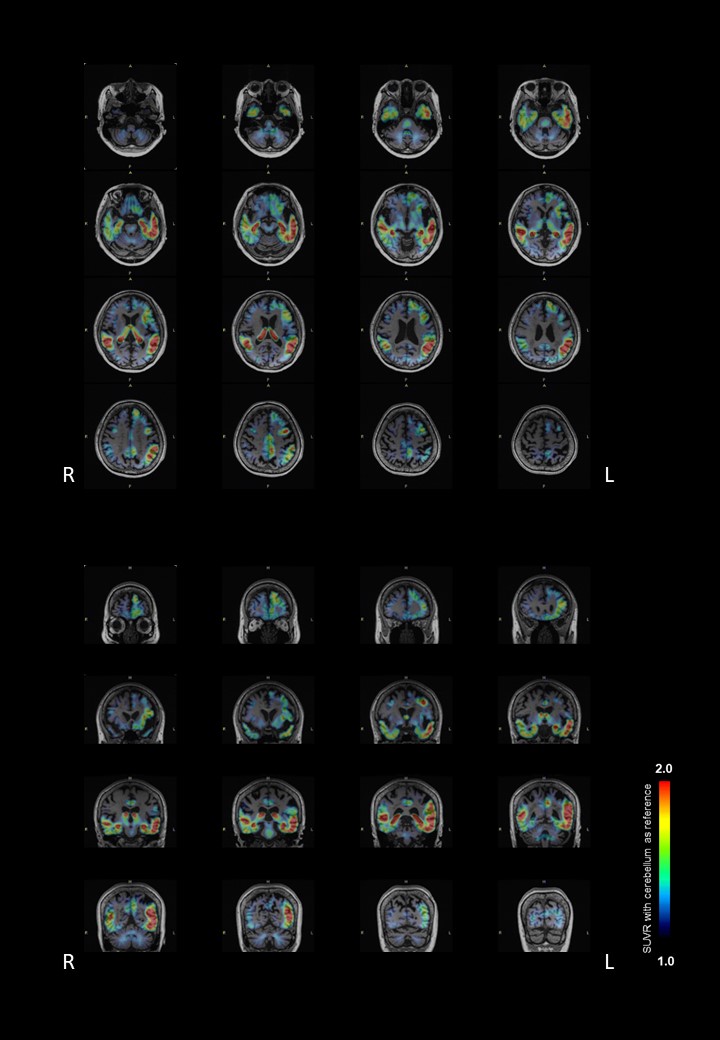

Supplement: Supplementary file 2 [file Image_1.jpg]
